# Metaphor Identification in Large Texts Corpora

**DOI:** 10.1371/journal.pone.0062343

**Published:** 2013-04-29

**Authors:** Yair Neuman, Dan Assaf, Yohai Cohen, Mark Last, Shlomo Argamon, Newton Howard, Ophir Frieder

**Affiliations:** 1 Department of Education, Ben-Gurion University of the Negev, Beer-Sheva, Israel; 2 Gilasio Coding, Ltd., Tel-Aviv, Israel; 3 Department of Information Systems Engineering, Ben-Gurion University of the Negev, Beer-Sheva, Israel; 4 Department of Computer Science, Illinois Institute of Technology, Chicago, Illinois, United States of America; 5 Synthetic Intelligence Lab, Massachusetts Institute of Technology, Boston, Massachusetts, United States of America; 6 Department of Computer Science, Georgetown University, Washington, D.C., United States of America; Max Planck Institute for the Physics of Complex Systems, Germany

## Abstract

Identifying metaphorical language-use (e.g., sweet child) is one of the challenges facing natural language processing. This paper describes three novel algorithms for automatic metaphor identification. The algorithms are variations of the same core algorithm. We evaluate the algorithms on two corpora of Reuters and the New York Times articles. The paper presents the most comprehensive study of metaphor identification in terms of scope of metaphorical phrases and annotated corpora size. Algorithms’ performance in identifying linguistic phrases as metaphorical or literal has been compared to human judgment. Overall, the algorithms outperform the state-of-the-art algorithm with 71% precision and 27% averaged improvement in prediction over the base-rate of metaphors in the corpus.

## Introduction

Human language comprises figurative and non-figurative dimensions. For instance, the use of the verb “fell” in the sentence “The coin fell into the sewer” is different from its use in the sentence “He fell in love”. In the first sentence, and according to the Oxford English Dictionary, the verb is used in its first sense of “move downward”. In the second sentence it is used in its extended metaphorical sense: “pass into a specified state”.

Differentiating figurative and non-figurative language-use may be highly important for a variety of applications that are based on natural language understanding. For example, giving a robot the order: “Give me the bottle” is totally different from giving the order: “Give me a break”. To obey the order, the robot must have a natural language understanding module that can differentiate between figurative and non-figurative language.

In this context, the justification for the current work may be stretched along several levels. At the minimal level, developing algorithms for metaphor identification is justified in order to improve the performance of different applications drawing on natural language understanding. For these applications it is necessary to differentiate figurative and non-figurative language as illustrated above.

From a wider theoretical perspective, we draw on the thesis that human behavior and reasoning is somehow mediated by metaphors and that metaphors people use may reflect their worldviews. In this context, algorithms for metaphor identification may be a first step for better understanding metaphors and the worldviews that they represent. Here we adhere to the minimal level only by keeping the general theoretical context in mind. However, we first start with the more general theoretical level framing and guiding our work.

The ability to differentiate figurative from non-figurative language is an important milestone in Natural Language Understanding because higher levels of human thinking may involve the use of metaphors, and metaphors may somehow reflect the way people think. This thesis emerged in Cognitive Linguistics, where the classical concept of metaphor as a rhetorical ornament has been replaced by the idea that metaphor is a psycholinguistic device for understanding one conceptual domain in terms of another conceptual domain (e.g., My lawyer is a shark) [1], [2]. In the sentence “My lawyer is a shark”, the Conceptual Metaphor involves the use of the ANIMAL category to describe the PERSON category. We are aware of the fact that Conceptual Metaphor Theory, as presented by George Lakoff has been criticized on several dimensions [3], [4]. Nevertheless, we adopt it as a starting point although the algorithms presented in the paper are independent on the theory’s validity.

In this paper, we aim to develop algorithms for metaphorical phrase identification. Identifying conceptual metaphors is another important challenge. However, this challenge is beyond the scope of the current paper.

It is argued that human thinking is not only highly metaphorical [2], but that metaphors mediate human behavior and reasoning [5]. While the simple causal influence of metaphors on reasoning and behavior may be questioned, the emerging empirical evidence of “language as context” for a variety of cognitive processes (e.g., [6], [7]) provides some support to the thesis that in a given context the use of different metaphors by the same subject may lead to different cognitive and behavioral responses. While [5] argument doesn’t go without critique, specifically in the context of the debatable relation between language and thought (see [8]), the language-thought debate is just one more justification for initiating the current study.

Metaphors have turned into an important object of research in fields ranging from psychology to natural language processing [5], [9]–[15]. In this context, a minimal condition for automated understanding of metaphorical language is the ability to differentiate between metaphorical and non-metaphorical language-use.

From the above example (My lawyer is a shark) we can see that the general definition of a conceptual metaphor is theoretically rather clear. However, in practice, the decision whether a given linguistic expression/phrase is metaphorical is far from trivial, as the boundary between the denotational basic meaning of a phrase and its extended metaphorical sense is fuzzy [16]. For instance, it is quite clear that a “sweet child” isn’t sweet in the same sense as “sweet chocolate”. In the first case, “sweet” is not used in its denotational “sugary” sense but in its extended metaphorical sense: pleasant. However, is “wise government” a literal or metaphorical language use? In this case, we will probably be less confident in making a decision.

One possible approach for resolving this difficulty is to adopt a commonsensical criterion for a metaphor and to consider the common use of a phrase as literal use and its violation as indicative of metaphorical use. However, the most frequent sense of a phrase cannot always be used to indicate its literal sense. To illustrate this point, we can examine the ten most frequent nouns co-located with the adjective “great”. We used the Corpus of Contemporary American English (COCA) [17], and following the norm of COCA used Mutual Information (MI) equal to or greater than 3 as a minimum criterion for significant statistical association between the words. Mutual Information (MI) is a quantity that measures the mutual dependence of two random variables. In our case, the mutual information of two words is a measure that indicates the degree to which two words are statistically associated. In the Corpus of Contemporary American English, Mutual Information of words A and B is calculated as follows:
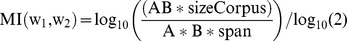
where

A = frequency of node word w_1_ (e.g., *purple*): 1262

B = frequency of collocate w_2_ (e.g., *color*): 115

AB = frequency of collocate near the node word (e.g., *color* near *purple*): 24

sizeCorpus = size of corpus (for instance 96,263,399)

span = span of words (e.g., 3 to left and 3 to right of the node word: 6)

log_10_ (2) = 0.30103

MI (purple, color) = 11.37 = log_10_ ((24*96,263,399)/(1262*115*6) )/.30103

By following the above procedure, we identified the top ten nouns located one lexical unit to the right of “great”. In all of these cases “great” was used in the sense of “important” (e.g., great deal, great job, great depression). However, the more basic “embodied” and literal sense of “great” is “very big, large scale”!

These results support the argument that using the common sense of the phrase as an indication of literal use is a wrong move. In fact, this is the main problem hindering the use of the “selectional preference” approach [18], [19] to metaphor identification, which considers violations of normative use as an indication of metaphorical language. Let us explain the meaning of “selectional preference” and its relevance for metaphor identification. “Selectional preference” describes the statistical “tendency” of words to co-occur with “selected’ or “preferred” words from certain semantic categories. This idea is presented by Light and Greiff [20] as follows: “Words in the same sentence stand in relationships with one another. For example, in *the person quickly ate the delicious sandwich*, the verbal predicate *eat* has *person* and *sandwich* as arguments. Similarly, *quickly* and *delicious* have as arguments *eat* and *sandwich*, respectively. These predicates have preferences for the semantic class membership of the arguments filling a particular role. For example, *eat* prefers, as its object argument, words from the semantic class of food and disprefers words from the semantic class of fluids.” (p. 269) We can simply explain “selectional preference” as the constraints or limitations of certain argument-predicate relations. This idea is further elaborated by Hoey [21] who suggests that all lexical items are primed by grammatical and selectional co-locational use. In the context of metaphor, we may argue that metaphorical language-use “violates” the constraints imposed by literal or figurative language use. For instance, if we know that the verb “Drink” selects nouns belonging to the semantic category of FOOD, then when we are asked to judge the sentence: “My car drinks gasoline”, we judge it to be metaphorical as the noun “gasoline”, which does not belong to the category of FOOD, violates the selectional preference of “Drink”.

Following Lakoff and Johnson [2], the criterion we chose in order to determine whether a phrase is metaphorical, is how close the word’s sense is to its embodied origins. Embodied origin is the way the concept is grounded in sensorimotor experience [2]. This is why “Big house” is a literal phrase and a “Big issue” is a metaphorical phrase. In its literal sense, “Big” points to a spatially large object but its metaphorical extended sense is “important”. A literal phrase can be traced to its embodied source while a metaphorical phrase is its extension. This is precisely the criterion we provide the annotators in this work for determining whether a phrase is literal or metaphorical.

A conceptual metaphor can be expressed in a variety of linguistic patterns. Following Krishnakumaran and Zhu’s [12] approach, we focus our work on three types of metaphorical phrases involving nouns. Those authors differentiate between type I, II, and III metaphors.

In a type I metaphor, a subject noun is associated with an object noun via a form of the copula verb “to be”, such as in the case of “God is a king”. For a type II metaphor, the verb is the focus of the metaphorical use representing the act of a subject noun on an object noun, such as in the case of “The war *absorbed* his energy”. Type III metaphors involve an adjective-noun phrase such as “sweet girl”.

It is clear that Krishnakumaran and Zhu’s typology of noun-based metaphors does not fully exhaust the wide spectrum and richness of metaphorical language. Therefore, we have no pretension that we will cover all forms of metaphors. Nevertheless, the typology offered by Krishnakumaran and Zhu identifies some of the most basic and important linguistic metaphorical structures, and therefore it is a heuristic that guides the development of algorithms for metaphor identification. Future studies will extend our results to other expressions and forms of metaphors. Here we present three algorithms for identifying the above types of metaphors in unstructured texts and evaluate the algorithms on two large corpora. As will be seen, these algorithms have outperformed the state-of-the-art algorithm for metaphor identification.

### Approaches to Metaphor Identification

Different approaches exist for the identification of metaphors ranging from Word Sense Disambiguation to the use of words’ categorization [9], [12] and clustering [22]. Let us explain some of these approaches starting with Word Sense Disambiguation (WSD). Many words in natural language have more than one sense. For instance, the word “Bread” may be used in the sense of FOOD or in its slang use as a synonym with MONEY. The ambiguity associated with word senses presents Natural Language Processing with many difficulties and Word Sense Disambiguation (e.g., [23]) is the field of research in which algorithms are developed to disambiguate the sense of a word in context. One approach to metaphor identification considers this task as analogous to word sense disambiguation and tries to “disambiguate” the sense of a given phrase in order to decide whether it is used in a literal or metaphorical sense. Along similar lines, we may use Word Categorization. Word categorization involves the classification of words into semantic sets. The use of word categorization is evident in the selectional preference approach we have described above. To classify noun semantics, in the current study we used the WordStat noun categorization based on WordNet, which classifies 69,817 nouns into 25 different categories, such as Artifact (e.g., Chair) or Food (e.g., Bread).

As there is no gold-standard for comparing metaphor identifications, it is rather difficult to evaluate the performance of different metaphor identification algorithms. However, to provide the reader with a wide perspective on the field we review some notable attempts relevant to automatic metaphor identification.

Hashimoto and Kawahara [24] discuss work on a similar problem, distinguishing idiomatic usage from literal usage. They also approach this as a classical word sense disambiguation task. Idioms are somewhat different from metaphors, in that the meaning of an idiom (e.g., *kick the bucket*) is often difficult to derive from the meanings of the component words, unlike most metaphors.

Nissim and Markert [25] use supervised learning to distinguish metonymic usage from literal usage. They take a classical WSD approach, learning a separate model for each target word. As with Birke and Sarkar [9], [26] and Hashimoto and Kawahara [24], the core idea is to learn to classify word usage from similarity of context.

Martin [27] presents a knowledge-based approach to interpreting metaphors. This approach requires complex hand-crafted rules, which are specific to a given domain (e.g., interpreting metaphorical questions from computer users, such as “How can I *kill* a process?” in an online help system). The knowledge base cannot handle words that are not hand-coded in its rules and a new set of rules must be constructed for each new application domain.

Dolan [28] describes an algorithm for extracting metaphors from a dictionary. Some suggestive examples are given, but the algorithm is not evaluated in any systematic way.

Mason [29] takes a corpus-based approach to metaphor. His algorithm is based on a statistical approach to discovering the selectional restrictions of verbs. It then uses these restrictions to discover metaphorical mappings, such as “Money flows like a liquid”. Although the system can discover some metaphorical mappings, it was not designed to distinguish literal and metaphorical usages of words.

Birke and Sarkar [9], [26] address the problem of metaphor identification as a classical word sense disambiguation task [30]. A model is learned for each verb independent of the other verbs. The problem with this approach is that it is limited and cannot handle a new verb without additional training.

Turney et al. [15] present the state-of-the-art algorithm for metaphor identification by comparing their results to those of Birke and Sarkar The rationale of their Concrete-Abstract algorithm is as follows. Type III metaphors, comprised of adjective-noun phrases as in “dark thoughts”, generally involve the use of a concrete concept (“dark”) to describe a more abstract concept (“thought”). Therefore, for the task of distinguishing between metaphorical and literal phrases, they used only one element: the abstractness rating of the noun in the phrase.

The basis of Turney et al.’s argument [15] was the hypothesis that if the noun in an adjective-noun phrase is relatively abstract, then the adjective is likely used in a concrete-embodied sense to explain the meaning of the noun, and therefore the phrase functions as a metaphor. For identifying type III and type II metaphors, they used a very simple measure–the abstractness level of the words. Drawing on a novel algorithm that rated the abstractness level of words, they measured the abstractness level of the noun in the case of type III metaphors and the average abstractness level of the nouns in the sentence in the case of type II metaphors. In other words, their algorithm is very simple. Given an adjective noun phrase such as “Sweet dreams”, the algorithm and the model take into account only one thing, which is the abstractness score of the noun in the phrase. Based on this abstractness score and the coefficient calculated in a Binary Logistic Regression model, a binary choice is made whether the phrase is metaphorical or not.

Tested on a list of 100 adjective-noun phrases, this algorithm resulted in an average of 79% accuracy [15]. Despite its impressive performance in terms of accuracy, it was recently shown by Assaf et al. [31] that at least with regard to type III metaphors, the Concrete-Abstract algorithm developed by Turney et al. has a blind spot, and its impressive performance is limited to cases where the adjective has a clear embodied base. This paper presents a preliminary examination of the Concrete Category Overlap (abbreviated as CCO) algorithm to be detailed below. The CCO was tested on a test set that is an extension of Turney et al.’s [15] test phrases and on a second test set that included adjective-noun pairs with nouns that have a lower abstractness level, such as in the word pair “Broken heart”. Assaf et al.’s [31] argument was that the Concrete-Abstract algorithm has a serious blind spot in dealing with such phrases. For instance, in the phrase “Broken heart” the noun “heart” is a relatively concrete object. Therefore, the Concrete-Abstract algorithm might judge the phrase as literal language-use. Indeed, it was shown that the Concrete-Abstract algorithm totally fails in dealing with such phrases, while the CCO algorithm performed well. It was therefore a preliminary test of the algorithm and a calibration of the algorithm’s parameters. In contrast, the current paper evaluates the CCO algorithm and its derivatives (CCO* and CCO**) on (1) complete sentences rather than on isolated phrases, (2) two large corpora, and (3) with regard to five target words.

### The Contribution of the Current Work

The contribution of the current paper in comparison to other works in the field can be briefly described along several dimensions.

1. In contrast to recent algorithms that are based on supervised learning, [22] our algorithms are rule-based and do not require a training corpus of metaphorical and non-metaphorical phrases.
2. While Shutova, Teufel, and Korhonen [22] have realized the importance of abstractness in metaphor identification, they have not developed or used any tool that measures the abstractness level of phrases. Our metaphor identification algorithms use such a tool developed by Turney et al. [15]. It must be emphasized, that in our algorithms the use of the abstractness measure is used as an indirect approximation of the noun’s embodied nature. This idea draws on [32] that have used this approach, with all the inevitable qualifications, for successfully measuring semantic relatedness.
3. Our algorithms integrate two approaches: selectional preference and abstractness-based metaphor identification, [15] a combination that has been shown [31] to solve the well-documented problems associated with the selectional preference approach and to outperform the state-of-the-art algorithm developed by Turney et al. [15].
4. In contrast with other works, our algorithms are lexicon and domain independent. Goatly [33], for instance, relies on a set of linguistic cues such as “metaphorically speaking”, whereas Mason [29] utilizes domain-specific selectional preferences. Our algorithms are not constrained by limited knowledge domains such as FINANCE or SPORT.
5. The algorithms are fully automatic and do not rely on hand crafted knowledge (e.g., [33]).
6. Our algorithms identify three major types of metaphorical phrases and are not limited to one type only, such as verb-based metaphors studied by [22].
7. Our algorithms were evaluated on two large corpora; for their evaluation we annotated a large number of phrases using several independent human annotators. For instance, in a recent paper by Shutova, Teufel, and Korhonen [22], the annotators received “78 randomly sampled sentences” (p. 41). In comparison, in the current study and for the Reuters corpus only, four annotators annotated *1378 phrases*.

We evaluated the algorithms on five target nouns: Governance, Government, God, Father, and Mother. The motivation for choosing these nouns was the interest in studying the metaphorical representation of government/governance. As government/governance may be metaphorically represented as extensions of parental images, what is called in the literature the symbolic father/mother, we included the target terms father, mother, and God–the ultimate symbolic father. The justification of using such a small number of words and the inevitable implications resulting from this choice are presented in the concluding section.

We present our algorithms for automated metaphor identification. As our algorithms emerged from the algorithm for the identification of type III (adjective-noun) metaphors, [31] we present it first.

### Identifying Type III Metaphors

As previously mentioned, the algorithm we have developed for identifying type III metaphors is titled the “Concrete Category Overlap” algorithm (CCO) [31]. Our starting point is Turney’s Concrete-Abstract algorithm. The CCO assumes, as do Turney et al. [15], that a metaphor usually involves mapping from a relatively concrete domain to a relatively abstract domain. However, it takes into account the importance of considering what those specific conceptual domains are. Literal use of a concrete adjective will tend to be more salient with regard to certain semantic categories of concrete objects and not others. For instance, in its literal use, the word “dark” may be associated with certain noun categories such as Physical Object (e.g., “table”) or Body Part (e.g., “skin”). *This notion leads directly to the CCO, which assumes that if the noun modified by an adjective or head noun belongs to one of the concrete categories associated with the literal use of the adjective, then it is probably literal and otherwise it is probably metaphorical.* In a sense, this algorithm combines the notion of measuring abstractness/concreteness and that of using selectional preferences, as has been well-explored in previous work on metaphor identification [18]. In other words, we adopt the idea that the selectional preference of the adjective for certain nouns may be indicative of metaphorical use, but first check the selectional preference of the adjective to concrete nouns only in order to establish its denotative literal sense. Therefore, while the Concrete-Abstract algorithm uses only the noun’s level of abstractness in order to determine whether the phrase is metaphorical, the CCO is much more complicated as it first identifies the most concrete nouns modified (i.e., selectively preferred) by the adjective and only then decides whether the target adjective-noun phrase violates this selectional preference. This hybrid approach overcomes the well-known issues of the pure selectional preferences approach, [34] in particular its tendency to over-generate metaphor hypotheses and be misled by common conventionalized metaphors. We first present a general overview of CCO, then its pseudo-code, and finally elaborate the algorithm’s fine details.

The CCO retrieves candidate adjective-noun phrases (*A, N*) with the target noun. It discards phrases identified in Wiktionary as idioms, or those where the adjective and/or noun have no dictionary definitions. Given the adjective-noun pair (e.g., strong government) the algorithm then works along the following lines. If the adjective A has a single dictionary definition then the phrase is labeled as LITERAL, since metaphorical usage cannot exist for one sense only. Else, the algorithm verifies that the noun *N* belongs to at least one WordNet category. If this is not the case, the algorithm cannot make a decision and stops. Otherwise, it identifies through a tagged n-grams corpus (COCA’s n-grams) up to θ_num_ nouns most frequently collocated with the adjective A, sorts them according to Turney’s abstractness scale [15] and chooses the κ most concrete nouns. These nouns are categorized using WordNet, and significant concrete categories having at least *θ_cat_* nouns each are selected. If the noun N does not belong to the any concrete WordNet category, return METAPHORICAL else return LITERAL. The pseudocode of CCO is presented below.

**Input:** An adjective-noun pair <*A,N*>.

1. If the adjective *A* has a single dictionary definition then return LITERAL, else
2. If the noun *N* does not belong to any WordNet category, then return UNDECIDED, else
3. Let **N**(*A*) be the θ_num_ nouns most frequently collocated with *A*, with mutual information of at least θ_MI_.
4. Let **N^C^** be the κ most concrete nouns in **N**(*A*).
5. Let **Cat**(*A*) be the set of all semantic noun categories containing at least θ_cat_ nouns in **N^C^**.
6. If *N* belongs to one of the categories in **Cat**(*A*), then return LITERAL, else return METAPHORICAL.

The basic idea is that an adjective is assigned to a set of semantic categories based on the most frequent concrete nouns that it modifies. We require these pairs to have minimum mutual information as well, to ensure that the nouns are closely associated with the adjective. Assaf et al. [31] identified these nouns by simple collocation in the Corpus of Contemporary American English (COCA), [17] setting the collocation lexical window to +1, θ_num_ to 1000, and θ_MI_ to 3. The 100 most concrete nouns were identified based on the abstraction scale developed by Turney et al., [15] which provides a degree measure from 0 to 1.0 with lower values being more concrete and higher values being more abstract.

To classify noun semantics, CCO uses the WordStat noun categorization based on WordNet (http://www.provalisresearch.com/wordstat/WordNet.html), which classifies 69,817 nouns into 25 categories, of which 13 are concrete categories (e.g., artifact). As CCO selects the most concrete nouns, it is expected that the words should be categorized only in these 13 categories. Based on statistics calculated from the most frequent 10,000 nouns in COCA, it was found that, on average, each noun is assigned to two categories. Therefore, if we randomly assign 100 concrete nouns to the 13 concrete categories we would expect, on average, 15.4 words in each category. CCO thus set **Cat**(*A*) to be all categories containing at least θ_cat_ = 16 nouns from **N^C^**. This helps avoid choosing categories that do not really represent literal use of the adjective *A*. The CCO algorithm has been evaluated on 682 phrases and outperformed the Concrete-Abstract algorithm [31]. Here we validate these findings by evaluating them on complete sentences drawn from the test corpora.

### Identifying Type II Metaphors

Verb-based metaphors are identified through a simple extension of CCO and this extension is named CCO*. For identifying the linguistic metaphor we used the three-word phrase T_P_ = <N_1_, V, N_2_>, where N_1_ is the subject noun, N_2_ is the object noun, and V is the verb linking them. Either N_1_ or N_2_ can be target nouns. For example, “The Fed ate my savings”, where the subject (“Fed”) is a target noun.

For deciding whether the phrase indicates metaphorical use, we first search Wiktionary for the definitions of the verb. If the verb has one definition only, then T_P_ is LITERAL, else we move to the next phase and search the n-grams corpus for the 1000 most frequent nouns collocated in a lexical window of +2 to the right of the verb and having MI≥3.

We rank the nouns on the abstractness scale and choose the 100 most concrete nouns. These nouns are categorized using the WordNet categorization, and the significant concrete categories are selected according to the same procedure applied in the CCO. Following the above example (“The Fed ate my savings”), we should identify the 100 most concrete nouns collocated with the verb [eat]. The categorization of these nouns will show us that the most dominant category is FOOD.

We also categorize the object noun (e.g., savings). If none of its nouns overlap with the categories of the 100 nouns associated with the verb, then we return METAPHORICAL. Else, we reduce the number of noun categories using the ConceptNet (http://csc.media.mit.edu/docs/conceptnet), which is a huge repository of common sense knowledge constructed by MIT. For example, searching the relevant category of savings we found that “Save is a fund”. If the most highly ranked category of the object noun is not included in the categories of the 100 nouns associated with the verb, then METAPHORICAL; else LITERAL.

The reason for this reduction procedure is illustrated through the following example. Assume we analyze the metaphor “She ate my heart”. The most concrete nouns collocated with “eat” can be categorized into two main categories: FOOD (e.g., ice cream) and ARTIFACT (e.g., cake). Heart can be categorized as BODY as well as ARTIFACT. Heart shares the ARTIFACT category with the category characterizing the most concrete nouns that are the objects of “eat”. However, it doesn’t mean that the phrase is literal. The pseudocode of CCO* is presented below.

**Input**: <N1, V, N2> (the verb V represents the act of the subject N1 on the object N2).

1. Identify the 100 most concrete object nouns associated with the verb V in a corpus.
2. Categorize the 100 nouns by using WordNet.
3. Categorize the object noun N2.
4. If none of the object noun categories overlaps with one of the categories of the 100 nouns associated with the verb, then return METAPHORICAL.
5. Find the main category of the object noun using ConceptNet.
6. If the main category is not included in the categories of the 100 nouns, then return METAPHORICAL; else return LITERAL.

### Identifying Type I Metaphors

A simple approach for type I metaphor identification is to compare the semantic categories of the nouns comprising the metaphorical expression, a strategy that was used by Krishnakumaran and Zhu [12]. For instance, the phrase “My lawyer is a shark” can be considered as metaphorical because the nouns “lawyer” and “shark” do not belong to the same category. A lawyer is a PERSON while a shark is an ANIMAL. This simple and intuitive approach suffers from a major problem underlying metaphor identification. This problem is the polysemous nature of words. If a noun is polysemous then it potentially belongs to several semantic categories. For instance, “Chicken” is both an ANIMAL and FOOD. Therefore, when trying to decide whether two nouns belong to the same category we find that they usually belong to several categories. Moreover, the fact that two nouns belong to the same category does not necessarily lead to metaphorical use. For instance, “My cat is a tiger” is a metaphor that may indicate the courage of my cat, despite the fact that both cat and tiger belong to the ANIMAL category. The algorithm we have developed for identifying type I metaphors aims to address this difficulty. As its final phase relies on similar logic to the CCO it is titled CCO**. The algorithm works along the following lines.

Given a target phrase T_P_ having the dependency representation nsubj(N_S,_N_T_), where N_S_ (subject) is the source domain and N_T_ (object) is the target domain, we first identify, through the WordNet categorization described above, the categories associated with the source and target nouns. If they do not overlap then the phrase is METAPHORICAL. Else, we move to the next phase that aims to disambiguate the noun’s categories.

The disambiguation is necessary as illustrated through the following example. In the phrase “My lawyer is a shark”, lawyer is categorized as a PERSON and shark as both ANIMAL and PERSON.

Again we use the ConceptNet and search it for the pattern “N_S_ is a C_S_” and “N_T_ is a C_T_”, where C_S_ and C_T_ are the categories identified at the previous phase. The categories identified at this phase are described as C_S_* and C_T_*. IF C_S_* ∩ C_T_* = Ø THEN T_P_ = ’METAPHORICAL’ else we move to the next phase.

Again, we identify the most frequent category associated with N_S_ and/or N_T_ according to ConceptNet ranking and search the n-grams corpus for the 1000 nouns that (1) are collocated in plus/minus 4 lexical units with N_S_ and N_T_, and (2) Having MI≥3. We scale the nouns according to abstractness level and choose the 100 most concrete nouns. These nouns are separately categorized for N_S_ and N_T_ according to the categories identified by the ConceptNet in the previous phase.

We choose the most dominant category associated with N_S_ and N_T_. The resulting categories are C_S_** and C_T_**, respectively. If none of the noun’s categories overlap, then it is METAPHORICAL else it is LITERAL.

**Input** <N1, N2> (a subject noun N1 associated with an object noun N2 via a copula verb).

1. Identify the categories of N1 and N2. If they do not overlap then return METAPHORICAL.
2. Find the main category of N1 and N2 using ConceptNet.
3. If the two main categories are different then return METAPHORICAL.
4. Identify the 100 most concrete nouns associated with N1 and N2 separately.
5. Categorize the 100 nouns associated with N1 and N2 separately.
6. If none of the nouns’ categories overlap with each other, then return METAPHORICAL; else return LITERAL.

## Data Sets

### A. The Reuters Corpus

Our first data set is the Reuters RCV1 dataset, [35] in which we search for texts including the five target words. The corpus size is around 3.9 M sentences. The final corpus we processed included 342,000 texts. In each text we identified the first sentence that included one of the target words. Our analysis focuses on unique sentences for the target words God (N = 536), Governance (N = 214), Father (N = 1923), Mother (N = 2253), and Government (N = 12800). Dead metaphors, or idioms, were removed from the analysis based on Wiktionary.

Each sentence was parsed using Stanford Dependency Parser [36]. Stanford POS Tagger [37] was used to identify word categories. Candidates for metaphor identification have been identified by searching for type I, II, and III phrases in the parsed sentence. Table 1 presents the distribution of target word instances across phrase (expression) types.

**Table 1:** The Reuters Corpus – Number of Annotated Expressions.

Expression Type			
Target word	I	II	III
Father	17	116	62
God	5	19	13
Governance	0	5	13
Government	86	277	613
Mother	12	81	59
**Total**	**120**	**498**	**760**

### B. The New York Times Corpus

The second corpus we used was the New York Times archive (1984), which contains around 70 M sentences. We searched the archive for documents containing each of the target words and extracted unique sentences (Governance = 5992, Government = 22,793, Father = 16,489, Mother = 15,786, God = 36,844). The metaphor annotation procedure was applied only to a subset of the target word occurrences due to the timing constraints of our experiment as detailed below.

### Evaluation

After a candidate phrase has been automatically identified by the NLP tools, the corresponding algorithm was run and the output was a binary decision whether a phrase is metaphorical or not. We ran our algorithms on CPU: i7-3820 CPU@ 3.60 GHz, Memory: 16 GB DDR3 1333 Mhz, Windows 7 Pro 64-bit with MS-SQL 2008 R2 Enterprise. Average running times for type I, II, and III metaphor identification were 0.3, 10.2, and 0.4 seconds, respectively.

To evaluate the algorithms’ performance, we compared their decision to human judgment. Four subjects, three of them native speakers of English and the fourth a medical student who has high proficiency in English, annotated the phrases to determine whether the phrase is used in its most salient embodied/concrete sense or in a secondary, extended metaphorical sense. For instance, in the case of “bitter lemon” the first embodied definition of the adjective “bitter” is “Having an acrid taste (usually from a basic substance)”. When asked to judge, as a training example, whether the phrase “bitter relations” is literal or metaphorical, the judges used the basic denotation of “bitter” to make a decision; as “relations” cannot have an acrid taste, the phrase is judged as metaphorical. Based on Turney et al. [15], the annotators received specific and detailed instructions for each metaphor type.

They were given the entire sentence in which the linguistic metaphor appears and in which the target word and its related lexical units were visually marked. Inter-annotator agreement, measured in terms of Cronbach’s alpha, was 0.78, 0.80, and 0.82 for type I, II, and III, respectively. The majority decision (i.e., 0 for literal and 1 for metaphorical) was used as a criterion to determine whether the use of the phrase is metaphorical.

When analyzing the decision accepted by the majority of annotators (at least 3 out of 4 agreed) for type I metaphors, we found that 76.6% of time they agreed on the classification of the phrase as literal and 19.3% of the time they agreed the phrase is metaphorical. In the case of type II metaphors, 48.6% agreed on literal categorization and 49.8% on metaphorical, and in the case of type III metaphors, 75.8% agreed on the literal and 15.2% on the metaphorical. These results indicate that despite the specific and detailed instructions given to the annotators, the decision whether a phrase is metaphorical is highly controversial. Intensive efforts are needed to train annotators even for a minimal agreement of whether real world phrases are metaphorical.

Table 2 presents the distribution of the phrases annotated as metaphorical/literal for the three metaphor types in the Reuters corpus.

**Table 2:** Annotators’ Decision – Reuters.

Expression Type			
Annotators’ decision	I	II	III
Literal	40 (33.3%)	242 (48.6%)	576 (75.8%)
Metaphorical	80 (66.7%)	256 (51.4%)	184 (24.2%)

Table 3 presents the distribution of the phrases annotated as metaphorical/literal for the three metaphor types in the NYT corpus. In this case, inter-annotator agreement was 0.80, 0.76, and 0.72, respectively.

**Table 3:** Annotators’ Decision – NYT.

Expression Type			
Annotators’ decision	I	II	III
Literal	129 (47.6%)	189 (63%)	272 (63%)
Metaphorical	142 (52.4%)	111 (37%)	160 (37%)

## Results

Results for the Reuters corpus are presented in Table 4. Due to the low prevalence of certain target words, we collapsed the results and present them across target words. The results were organized in a 2 by 2 matrix where the algorithm’s prediction (metaphor vs. -metaphor) is compared to the annotators’ decision (metaphor vs. -metaphor). The a priori measure is the proportion of metaphorical phrases in the corpus. In this context, the meaning of Precision is the proportion of correct positive results divided by the number of all returned positive results. In other words, it is the number of true metaphors; that is, phrases identified by the annotators as metaphorical, out of the total number of phrases identified as metaphorical by the algorithm. The Recall is the proportion of phrases identified by the algorithm as metaphorical out of the total number of true metaphors. The average precision is 71%, and the improvement in prediction over the a priori probability for types I, II, and III is 17.2%, 24.7%, and 30.2%, respectively. The *average improvement is 24%*. This means that a random guess whether a phrase is metaphorical would have been based on the metaphors’ prevalence in the corpus. However, the probability of identifying a metaphor given the algorithm’s decision that a metaphor has been identified would have improved the precision by 24%. The recall results show that we could successfully retrieve between 43% and 97% of actual metaphors.

**Table 4:** Results – Reuters.

Expression type	A priori probability	Precision	95% CI	Recall
I	66.7%	83.9%	75–90%	97.5%
II	51.4%	76.1%	70–80%	82%
III	24.2%	54.4%	45–61%	43.5%

We compared the performance of CCO’s algorithms to the state-of-the-art results gained by Concrete-Abstract (here abbreviated as Con-Abs) algorithm of Turney et al. [15]. For type I metaphors we used the abstractness levels of N1 and N2. For type II metaphors we measured the averaged abstractness level of the nouns in the sentence. Regarding type III metaphors, Con-Abs failed to produce any results. Results for types I and II are presented in Table 5 where CCO is used as a generic term for CCO* and CCO**.

**Table 5:** Comparative Results – Reuters.

Expression type	Precision CCO	Precision Con-Abs	Recall CCO	Recall Con-Abs
I	83.9%	76.53%	97.5%	76.53%
II	76.1%	63.87	82%	67.2%

We can see that our algorithms outperformed the results of Concrete-Abstract. Combining CCO’s decision and Con-Abs’ in a Binary Logistic Regression Analysis has not improved the precision but only the recall to 95%.

Table 6 presents the results for the NYT corpus.

**Table 6:** Results – NYT.

Expression type	A priori probability	Precision	95% CI	Recall
I	52.4%	84.1%	77–89%	85.9%
II	37%	62%	54–69%	83.8%
III	37%	69.8%	63–75%	88.1%

The average precision is 72%, which is quite similar to the average precision found in the Reuters corpus. The improvement in prediction over the a priori probability for types I, II, and III is 31.7%, 25%, and 32.8%, respectively. *The average improvement is 29.8%*. More than 80% of all metaphorical phrases of types I–III could be successfully retrieved by our method.

Again comparing the performance of our algorithms to Con-Abs, we found that Con-Abs failed to produce any results for type III metaphors. Results for types I and II are presented in Table 7.

**Table 7:** Comparative Results – NYT.

Metaphor type	Precision CCO	Precision Con-Abs	Recall CCO	Recall Con-Abs
I	84.1%	65.6%	85.9%	72.5%
II	62%	50%	83.8%	5%

We can see that our algorithms outperformed Con-Abs. In this case, combining the prediction of CCO algorithms and Con-Abs has not resulted in any significant change in precision or recall.

### Conclusions

The exponential increase in the amount of textual information has not been proportionally accompanied by computational tools that may turn this “semantic sphere” [38] into meaningful and usable patterns. As argued by Gruber, [39] we have not yet learned how to turn “collected intelligence” into “collective intelligence”. To address this challenge, one must develop sophisticated NLP algorithms inspired by human intelligence.

To the best of our knowledge, the current paper presents the most comprehensive study of metaphor identification in terms of scope of metaphorical phrases and annotated corpora size. The rule-based algorithms have shown a significant predictive value that is resilient across two different corpora. However, being limited to five target words, the results should be cautiously used, as variability of identification is evident across words and corpora. This point should be further explained. Originally, we started our analysis with a larger list of target words. However, searching the corpora we immediately noticed the extremely low prevalence of metaphors in a corpus. Metaphorically speaking, searching for metaphors was like searching for a needle in a haystack. This low prevalence deductively implies searching and processing bigger portions of the haystack/corpus in order to find a minimal number of metaphors for annotation. In the tradeoff between adding more target words and getting a very limited number of cases for analysis, or limiting our search to five target words, we have chosen the second option, which increases the statistical power of our analysis. The aim of the paper was to provide algorithms for metaphor identification and in this context “internal validity” was more important than “external validity” seeking to extend the algorithms’ performance beyond the specific sample. Our decision was also based on the realization that the population of words is so big that even multiplying our sample by a factor of three would not have resulted in a representative sample of words in natural language. We must stress the fact that our work involves the most comprehensive annotated corpora in the field but that we modestly acknowledge the limits of a single study, extensive as it may be, and the need for further studies with more words involved.

The algorithms’ reliance on the concreteness-abstractness of a target word may be improved through knowledge gained in neuroscience concerning the embodied base of words [40], [41]. The translation of this emerging knowledge into metaphor identification algorithms is far from trivial but seems to be a promising direction specifically when combined with empirically grounded norms of the neural basis of metaphors. [42].

Several difficulties are identified through the analysis. First, the metaphor identification is dependent on the identification of candidate expressions by the dependency parser, which is not error free. In this sense, the performance of a given parser *sets an upper limit* on the algorithm’s performance. For example, evaluating structured phrases (e.g., adjective-noun pairs) rather than free-text sentences, the CCO produced 80% precision, [31] in contrast with an average of 62% precision found in the current study. Future studies should therefore rely on better tools for parsing.

Another difficulty evident in metaphor identification research is an objective criterion for metaphor identification. The relatively low inter-annotator agreement in some cases is an indication that there must be a better procedure for deciding whether an expression is metaphorical or literal and that annotators should be intensively trained in advance to reach consensual decisions, possibly by using a reliable dictionary that differentiates between the embodied sense of a word and its extended metaphorical sense.

The metaphor identification algorithms proposed in this paper are generic, and they can be extended to other languages provided sufficiently accurate linguistic resources and tools. We are currently evaluating them on three additional languages with initial results indicating the applicability of the algorithms to those languages.
